# 545. Remdesivir And Obeldesivir Retain Potent Activity Against SARS-CoV-2 Omicron Variants

**DOI:** 10.1093/ofid/ofad500.614

**Published:** 2023-11-27

**Authors:** Lauren Rodriguez, Jiani Li, Dong Han, Ross Martin, Jasmine Moshiri, Nadine Peinovich, John P Bilello, Jason K Perry, Charlotte Hedskog

**Affiliations:** Gilead Sciences, Inc., Foster City, California; Gilead Sciences, Inc., Foster City, California; Gilead Sciences, Inc., Foster City, California; Gilead Sciences, Foster City, California; Gilead Sciences, Inc., Foster City, California; Gilead Sciences, Inc., Foster City, California; Gilead Sciences, Inc., Foster City, California; Gilead Sciences, Inc., Foster City, California; Gilead Sciences, Inc., Foster City, California

## Abstract

**Background:**

Remdesivir (RDV), a nucleotide analog prodrug that targets the viral RNA-dependent RNA polymerase Nsp12, is approved to treat COVID-19 in hospitalized and nonhospitalized patients. Obeldesivir (ODV), an oral mono-5’-isobutyryl ester prodrug that is metabolized into the same active triphosphate as RDV, is being evaluated in Phase 3 clinical trials. The antiviral activity of RDV and ODV against previous Omicron subvariants (BA.1 to BQ.1.1) was maintained with respect to the ancestral WA1 strain. We assessed RDV and ODV antiviral activity against recent Omicron subvariants BF.7, BQ.1, XBB.1.5, CH.1.1, and XBF using clinical isolates and/or site-directed mutants (SDMs).

**Methods:**

The prevalence of Nsp12 substitutions in Omicron subvariants was evaluated by analysis of *Global Initiative on Sharing Avian Influenza Data* (GISAID) EpiCoV database sequences. Structural analysis of identified substitutions was conducted on a prior cryo-electron microscopy-based model of the replication-transcription complex. Antiviral activity of RDV and ODV against subvariant clinical isolates was assessed by nucleoprotein ELISA in A549-hACE2-TMPRSS2 cells and by SDMs in the replicon system

**Results:**

Genomic analysis of > 2 million Omicron subvariant sequences revealed unique substitutions in Nsp12 vs WA1. No new defining substitutions in Nsp12 were found compared with earlier Omicron variants. The defining substitutions (≥ 75% of sequences) included P323L (all), Y273H (BQ.1), and G671S (XBB.1.5, CH.1.1, XBF). Less prevalent substitutions were observed with frequencies from 1-3.5%: L247F, T248I, V257F, N507I, A529V, F694Y; none had direct interaction with the incoming RDV nucleotide triphosphate or the viral RNA, except N507I. Phenotyping of individual substitutions using SDMs showed no loss of RDV or ODV susceptibility (< 1.2-fold change in EC_50_ vs WA1). Evaluation of CH.1.1 and XBF by introducing variant-defining mutations into the replicon showed no change in in vitro susceptibility (< 1.8-fold change). Phenotyping of clinical isolates of BF.7, BQ.1, XBB.1.5, and CH.1.1 indicated no loss of RDV or ODV in vitro antiviral activity (< 1.3-fold change).

**Conclusion:**

RDV and ODV retained potent in vitro antiviral activity against all tested Omicron subvariants with potencies comparable to WA1.

**Disclosures:**

**Lauren Rodriguez, PhD**, Gilead Sciences, Inc.: Employee|Gilead Sciences, Inc.: Stocks/Bonds **Jiani Li, PhD**, Gilead Sciences, Inc.: Employee|Gilead Sciences, Inc.: Stocks/Bonds **Dong Han, MS**, Gilead Sciences, Inc.: Employee|Gilead Sciences, Inc.: Stocks/Bonds **Ross Martin, PhD**, Gilead Sciences, Inc.: Employee|Gilead Sciences, Inc.: Stocks/Bonds **Jasmine Moshiri, PhD**, Gilead Sciences, Inc.: Employee|Gilead Sciences, Inc.: Stocks/Bonds **Nadine Peinovich, MPH**, Gilead Sciences, Inc.: Employee|Gilead Sciences, Inc.: Stocks/Bonds **John P. Bilello, PhD**, Gilead Sciences, Inc.: Employee|Gilead Sciences, Inc.: Stocks/Bonds **Jason K. Perry, PhD**, Gilead Sciences, Inc.: Employee|Gilead Sciences, Inc.: Stocks/Bonds **Charlotte Hedskog, PhD**, Gilead Sciences, Inc.: Employee|Gilead Sciences, Inc.: Stocks/Bonds

